# Differences in Dental Implant Survival between Immediate vs. Delayed Placement: A Systematic Review and Meta-Analysis

**DOI:** 10.3390/dj11090218

**Published:** 2023-09-15

**Authors:** Rishi Patel, Cemal Ucer, Simon Wright, Rabia S. Khan

**Affiliations:** 1 ICE Postgraduate Dental Institute and Hospital, University of Salford, 24 Furness Quay, Salford M50 3XZ, UK; rishipatel90@hotmail.com (R.P.); cemalucer@me.com (C.U.); profwright@glencairndental.co.uk (S.W.); 2 Department of Medicine, University of Lancaster, Lancaster LA1 4YR, UK

**Keywords:** immediate implant placement, survival, dental implants, delayed implant placement

## Abstract

Objectives: To compare the impact of immediate and delayed implant placement upon the survival of implants and to investigate the differences in implant survival between immediate and delayed placement in adults. Methods: A search for the relevant literature was performed using the databases of CENTRAL, MEDLINE and Scopus. The studies found were limited to publications between 2014 and 2022, written in the English language, peer-reviewed, and were randomised trials or comparative studies. The quality of the evidence was assessed using the Cochrane Risk of Bias 2.0 and Risk of Bias in Non-randomised Studies—of Interventions appraisal tools and implant survival, and the primary outcome was meta-analysed using RevMan v.5.3. Results: A total of 10 studies were eligible for inclusion, including six randomised controlled trials and four non-randomised comparative studies. Five of the six randomised trials observed a low risk of bias, while the comparative studies had a moderate-to-serious risk of bias. The search strategy resulted in 341 implants placed immediately into fresh extraction sites (332 survived, 97.4%) and 359 implants inserted into delayed sites (350 survived, 97.5%). Conclusion: The meta-analysis demonstrated that there was no significant difference in the implant survival rates between immediately placed implants and implants placed using a delayed timing protocol (risk ratio 0.99; 95% CI 0.96, 1.02, Z = 0.75, *p* = 0.45). However, the detailed analysis showed that slightly more implant failures happened in the immediate dental implant placement group, with survival rates in some studies ranging between 90 and 95%, while the delayed placement group had survival rates of more than 95%.

## 1. Introduction

Dental implants are an increasingly popular form of treatment among dental practitioners due to their ability to provide a desirable fixed functional and aesthetic outcome that closely resembles the properties of natural dentition [1]. However, some of the main reservations about implants reported across the literature and observed in clinical practice have been the techniques of implant placement, timing of placement, differences in survival rates, design type and other clinical factors [2]. 

The differences in implant survival have been reported to be significant between patients who receive implants at the time of (immediate—when a dental implant is placed immediately after a tooth extraction) and at a time following (delayed—when the placement of an implant is performed 3–4 months after an extraction) tooth loss, with some evidence showing that the differences in survival can be as high as 10% [2,3]. The placement of implants at the time of dental extraction (immediate placement) has become a common approach for dentists globally due to recent evidence showing that immediate placement is associated with the following successful osteointegration of the implant: reduction in the number of dental procedures which patients undergo and decrease in the duration of treatment compared to delayed placement [4]. Most of the early literature exploring the differences in survival following immediate and delayed implant placements has comprised predominantly non-controlled research. Evian et al. [2], in a retrospective observational design study, analysed data from 149 implants over 943 days. The survival rates between patients who received immediate and delayed placements were similar (78.2% vs. 81.2%). However, due to the small sample size in both groups, certainty in the survival rates may be unreliable. Furthermore, the low survival rates for both implant protocols are likely due to high incidences of periodontal disease among the cohorts, which is a problem known to affect osseointegration and implant stability. In fact, Veitz-Keenan and Keenan et al. found that implant survival was more favourable among patients lacking periodontitis (92–100%) as opposed to those with periodontitis (79–100%). A follow-up period of 1.2 to 16 years in those studies provided useful evidence that periodontal disease is a key factor in implant failure; however, the authors did not expand on the results of the immediate versus delayed implant groups. As a result, uncertainty has persisted over the years with regard to the value of such timing protocols, particularly for patients with medical and dental comorbidities.

However, immediate implant placement is not universally accepted due to conflicting evidence showing that the insertion of implants into fresh extraction sites can reduce the rate of osteointegration and, in some cases, result in poor satisfaction among patients, a revision of the procedure and, ultimately, the failure of the implant [5]. Despite this, various studies have shown that the differences between implants placed immediately or delayed protocols upon aesthetic and functional results are negligible, and thus the focus within the field has shifted towards evaluating the impact upon implant survival [6,7,8,9]. A review of important evidence evaluating the impact of implant placement timing upon survival has been explored in the following literature review for contextual and justification purposes. Thus, the aim for this systematic review and meta-analysis is to provide an updated evaluation of the survival rates between immediate versus delayed implant placements. In addition, another aim is to determine whether any ongoing knowledge gaps persist regarding the factors influencing implant survival, such as treatment timing, smoking, periodontal disease and medical conditions. 

The evidence presented suggests that delayed implant placement may result in a slightly higher survival rate than immediate implant placements, at the potential risk of reduced aesthetic results and increasing treatment time and thus patient discomfort. However, due to the significant differences in survival rates reported, as well as the pre-existing evidence being affected by low sample sizes and methodological issues, there remains uncertainty about the most optimal approach in terms of the timing of implant placement. The rationale for this systematic review and meta-analysis is to provide an updated evaluation of the survival rates between immediate versus delayed implant placements. 

## 2. Materials and Methods

A central research question was developed to guide the methodology and methods of the review and to maintain focus upon the specific topic of interest [10]. The question was derived following the identification and incorporation of the PICO (population, intervention, comparator and outcomes) components shown in Table 1 [11]. The research question is summarised as follows: what are the differences in implant survival between immediate versus delayed placement in adults aged more than 18 years? The protocol was registered with PROSPERO CRD46209599271.

Rationale for reviewing the literature, in addition for the need to update the prior review of Chrcanovic et al. (2015) [12], was attained following a scoping search for any similar reviews, which were not identified; the search was performed using MEDLINE. A primary study was not amenable to determining the overall impact of immediate versus delayed dental implant placements due to funding limitations, lack of resources and the methodological challenges previously noted. Objectivity in the systematic review methods and reporting was supported by compliance with the Preferred Reporting Items for Systematic Reviews and Meta-Analyses (PRISMA) criteria [13]. 

### 2.1. Search Strategy

A search for evidence needed to address the research question was carried out using online electronic databases in November 2022 as the primary information source due to the ease, power and efficiency of permitting literature searches (Aveyard, 2018). The databases searched included the Cochrane Central Register of Controlled Trials (CENTRAL), MEDLINE and Scopus. A ‘snowballing’ citation method was used to optimise the value of this process, a technique that has been found to enhance search precision [14]. In addition, a free-text search for grey literature and non-journal publications was performed via Google Scholar [15]. Data were extracted by R.P into Microsoft Excel, which included the main features of each article including title, author(s), date, country, study methodology and main findings/results. The data extraction process was cross-checked by all authors using a standardized data extraction method to reduce selection bias. Once the final articles were selected, data were extracted using tables to formulate further details about the study process and study outcomes. 

Search terms were developed in accordance with the PICO elements of the review question, and these were then supplemented with a range of other synonymous and similar terms that were recognised following a preliminary review of the relevant literature [15]. The search strategy has been summarised in Table 2. 

### 2.2. Inclusion and Exclusion Criteria

As summarised in Table 3, the inclusion and exclusion criteria were applied after the removal of duplicate studies and the processes of title/abstract screening and full-text screening. 

### 2.3. Quality Assessment

The methodological quality of studies included in the review was subject to critical appraisal, guided by the Cochrane Collaboration’s Risk of Bias in Non-randomised Studies (ROBINS-I) and Risk of Bias (RoB) 2.0 tools via Review Manager 5.4.1 which is a Cochrane software, New York, NY, U.S. These tools were selected for being specific to the designs restricted for inclusion in this review and for assisting in producing all important issues of internal (risk of bias) and external (generalisability) validity [16,17]. The importance of appraising evidence and deriving overall judgements of quality should not be undermined in view of the value and influence of evidence-based principles upon ongoing dental practice, guidelines, policy and ongoing research. In the absence of a quality assessment, certainty and confidence in developing recommendations for ongoing dental implant practice would be unclear and misinformed, which are issues that would ultimately render the review of little value. 

### 2.4. Data Extraction

Data required for critical appraisal, evidence management and the production of results were obtained in accordance with the objectivity expected of PRISMA-based systematic reviews [11]. Such rigour was essential to minimising the risk of extraction errors, which have been found to occur with high prevalence and lead to biased outcome effects in previously published reviews [18]. The extraction proformas used in this review were taken from the Cochrane Handbook for Systematic Reviews, which were specific to the randomised and observational designs of evidence included to ensure all important data were extracted and available for appraisal and analysis [19]. Furthermore, the proformas were modified prior to extraction to accommodate aspects of the concept of interest. 

### 2.5. Data Analysis

The technique of statistical meta-analysis was used to collectively analyse the data concerning the primary outcome of implant survival. This was due to this method allowing an objective analysis of specific outcomes and generate an overall outcome effect related to an intervention of interest [20]. Implant survival or failure was analysed as a dichotomous outcome and used to create the forest plots depicting intervention effects based on the pooling and analysis of data. Studies that had implant survival data that were unclear or unreported were excluded from the meta-analysis. 

When inter-study heterogeneity was detected with a statistical significance defined as a usual alpha > 0.05, an inverse variance method was used to generate the pooled analysis using a random effects model. If inter-study heterogeneity was limited, a fixed-effects model would have been used to find the inverse variance method [21]. The following accepted categories were used to determine inter-study heterogeneity: 25%—low, 50%—moderate, and 75%—high. However, statistical significance was used to guide the mode of analysis as previously discussed [11]. 

The rationale for the inverse variance method was built on the idea that studies with a large sample size tend to have smaller variances of effects from the mean and therefore are given greater weight in pooled analyses. Smaller studies are given lesser weight for the opposing reasons [22]. Accordingly, this helps to derive more precise confidence in the outcomes given that the studies are weighted according to the degree of standard error [22]. In addition, the meta-analysis was accompanied by a funnel plot, as this shows the effect size against standard error, to extract any publication bias or other biases developing due to issues of sample size [23]. Review Manager v5.3 software provided by the Cochrane Collaboration for systematic reviewers was used to perform meta-analysis [16]. 

## 3. Results

### 3.1. Study Selection

**Figure 1:**
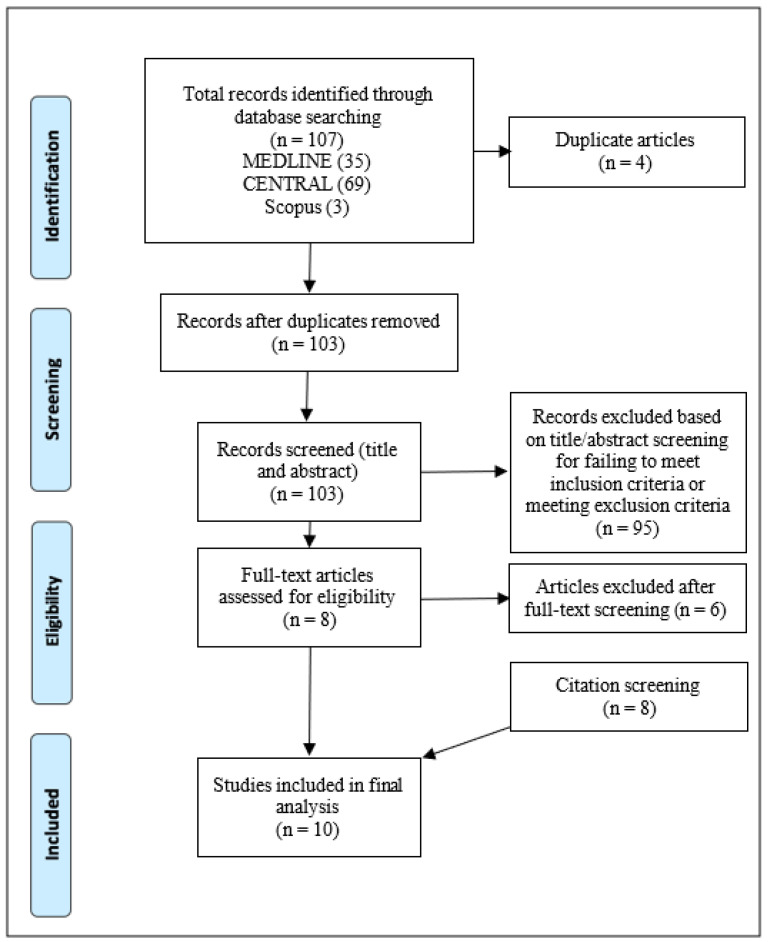
Process of evidence selection using PRISMA reporting diagram.

The search for the relevant literature using the strategy defined in Section 2.3. retrieved a total of 107 records. Among these articles, four duplicates were detected and discarded, so that only unique studies were subject to the filtering steps of title/abstract and full-text screening. The remaining 103 studies were title/abstract screened; the application of the inclusion and exclusion criteria resulted in the exclusion of 80 articles, which failed to meet one or more of the inclusion criteria or met at least one exclusion criterion. The residual studies (n = 8) underwent full-text screening, which also led to the removal of six studies. The specific reasons for exclusion at the full-text stage of filtering are included in Figure 1. Therefore, a total of 10 studies were eligible for the review. The characteristics of the studies are described in the following subsection. 

A summary of the filtering process is illustrated in Figure 1.

### 3.2. Study Characteristics

Among the 10 studies included in this review, all authors sought to investigate the impact of immediate versus delayed implant placement upon the survival of implants over varied time periods [24,25,26,27,28,29,30,31,32]. 

A summary of the main evidence characteristics of the cited studies is shown in Table 4. The research designs included six randomised controlled trials [24,25,26,29,30,31], a non-randomised comparative study [27], two prospective cohort studies [28,33] and one retrospective cohort study [32]. 

The populations included in the studies are included in Table 1 and were generally balanced between the intervention and comparator groups. They are as follows: adults requiring implants in the posterior mandible [24], predominantly premolar and molar regions [25], maxillary region from second premolar to second premolar region [26], all anterior and posterior dental regions [27], implants in posterior regions mainly in the mandible [28], implants in the premolar or molar sites [29], implants in the anterior maxilla after single-tooth extraction [33], implants in bony defects within the anterior aesthetic zone [30], implants in the anterior and premolar regions following single-tooth extraction [31] and implants inserted in the maxillary and mandibular regions of head and neck surgery patients [32]. All studies reported the outcome of implant survival at said time points, while most also explored other outcomes of importance, which are summarised in Table 5. 

### 3.3. Quality Assessment

Using the approach to critical appraisal via the ROBINS-I tool for non-randomised studies and the RoB 2.0 tool for randomised controlled trials, judgements on the methodological quality were reached. A summary of the quality assessment outcomes is shown in Table 6.

### 3.4. Meta-Analysis and Key Findings

#### Immediate versus Delayed Placement upon Implant Survival

Among the 10 studies eligible for review, all were agreeable to the pooling and meta-analysis of data regarding the difference in implant survival between immediate and delayed implant placement groups.

The implant survival rates for the immediate and delayed implant groups in each study were reported as follows (survival rates are given, respectively, relative to immediate and delayed placement): 94.7% vs. 100% (Bömicke et al. [24]), 95.9% vs. 100% (Cucchi et al. [25]), 96.3% vs. 100% (Esposito et al. [26]), 97.8% vs. 98.1% (Hakobyan et al. [27]), 100% vs. 96.9% (Han et al. [28]), 100% vs. 100% (Malchiodi et al. [29]; Slagter et al., [30]), 93.8% vs. 100% (S. Raes et al. [33]), 98.3% vs. 100% Tonetti et al. [31] and 97.4% vs. 90.5% (Woods et al. [32]).

Therefore, implant survival for implants placed immediately following tooth extraction only exceeded that of delayed placement in two studies (mean difference in survival in those two studies: 5.0%), although one observed a moderate risk of bias, Han et al. [28], and the other was based on patients with head and neck pathologies that would have directly influenced implant viability and longevity, Woods et al. [32]. In contrast, delayed implant placement was associated with superior implant survival in most of the trials (n = 6) included in this review [24,25,26,27,31,33]. Comparable survival rates between immediate and delayed implant placement were observed in two studies [29,30], but this may be explained by the limited follow-up period of one year and the relatively small sample sizes included in the analyses, as well as the studies excluding smoking and parafunctional habits with regard to their eligibility criteria. Furthermore, gender was not specified, and an exploration of these factors might reveal interesting facts about survival rates of implants.

**Figure 2:**
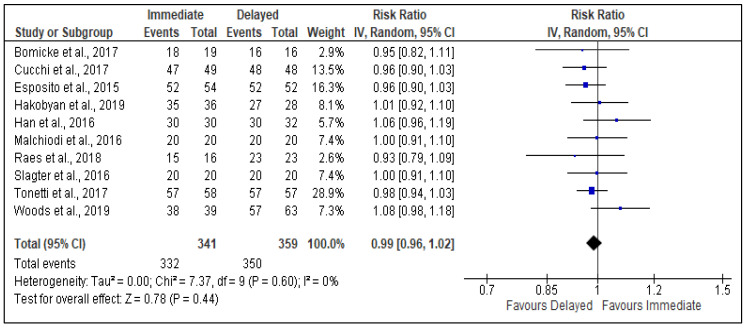
Forest plot showing pooled meta-analysis of the effect of immediate as opposed to delayed implant placement on implant survival [9,24,25,26,27,28,29,30,31,32].

The pooled analysis (fixed-effects model) is shown in Figure 2, which shows that there was no difference in the risk of implant survival/failure between the immediate and delayed implant placement groups (risk ratio 0.99; 95% CI 0.96, 1.02), which therefore did not reach statistical significance (Z = 0.78, *p* = 0.44). The level of heterogeneity across the included studies was low (I^2^ = 0%), and thus, the *p* value indicated that inter-study heterogeneity was not statistically significant (*p* = 0.60). Overall, the meta-analysis and the limited heterogeneity between studies suggest that there is high confidence and certainty in the reported outcome effect, as there is likely to be minimal deviation in said effect related to any biases detected among the informing studies.

**Figure 3:**
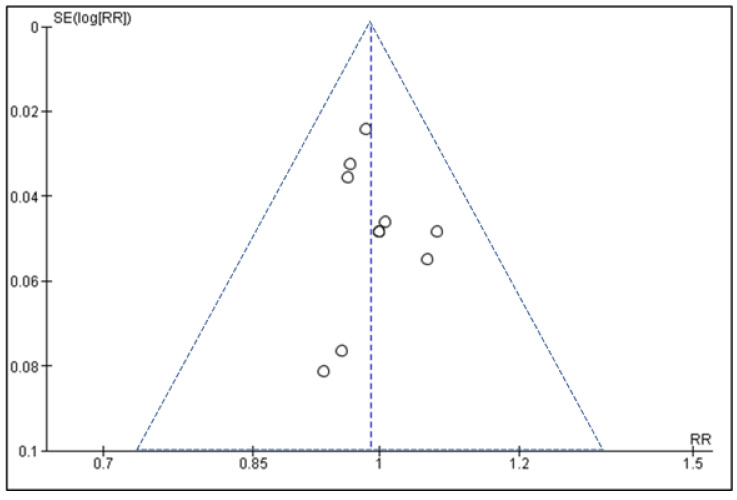
Funnel plot showing the relationship between risk ratio and standard error for the pooled meta-analysis.

The funnel plot is shown in Figure 3, and based on the meta-analysis previously described, it shows some minor asymmetry, suggesting that there could be a small risk of publication bias but one that would be unlikely to meaningfully alter the pooled outcome effect.

## 4. Discussion

This systematic review and meta-analysis aimed to investigate the impact of immediate versus delayed implant placement on implant survival, addressing existing uncertainties

This review builds upon a prior systematic study by Chrcanovic et al. [12] by incorporating the latest evidence published within the past seven years

Through a detailed search for relevant studies, a total of 10 articles were eligible for inclusion and were limited to the English language, including six randomised controlled trials and four non-randomised studies [26,27,28,29,30,31,32,33]. All the studies included in this review were relevant in terms of pooling data regarding implant survival and meta-analysis. The meta-analysis demonstrated that there was no significant difference in the rate of implant survival between immediate and delayed implant placement groups (risk ratio 0.99; 95% CI 0.96–1.02, *p* = 0.45). The funnel plot, however, showed a small asymmetry and potentially a risk of bias in some of the studies included in the analysis.

However, the data collection for implant survival across the ten studies showed that delayed implant placement offered higher survival rates compared to immediate implant placement

Delayed implant placement often resulted in survival rates of 100%, with a few low survival rates in certain studies among the delayed implant cohort being recorded as a possible result of underlying methodological issues. In a couple of studies, the implant survival was 100% across both the immediate and delayed placement groups, although these findings were seen among small sample sizes.

Other important findings included that there was a higher association of implant failures in placements in type C sockets of the Smith and Tarnow [34] classification and in patients with pre-existing medical conditions. Only one study out of ten found that complications were significantly more prevalent in the immediate implant placement group. However, the evidence across the studies showed that there tended to be greater probing depths and crestal bone loss among immediately placed implants, although implant stability (as measured by ISQ) and aesthetic outcomes were comparable between the immediate and delayed implant groups.

Overall, the findings of this review suggest that there is no significant statistical difference between immediate and delayed implant placement in terms of implant survival or other complications. However, in general, delayed placement may offer a slight survival benefit in selected cases, such as patients with other existing dental and medical comorbidities. The survival criteria used by the authors were subjective, and RCTs should be conducted with objective survival criteria such as those proposed by Albrektsson et al. or Misch et al. [35]. Therefore, the lack of objective survival criteria could explain the differences in the outcomes of the studies published.

A vast body of previous literature has compared the differences in implant survival among patients who received immediate and delayed implant placement. In the most pertinent evidence source, Chrcanovic et al. carried out a systematic review and meta-analysis of 73 studies that analysed data for more than 8200 dental implants placed. The meta-analysis revealed that the relative risk of implant failure among the immediate implant placement group was significantly higher compared to the delayed implant placement group (RR 1.58, 95% CI 1.28–1.95, *p* > 0.0001). This contrasts with the findings of this meta-analysis, which did not show a statistical difference in survival between the same intervention and comparator conditions (RR 0.99; 95% CI 0.96–1.02, *p* = 0.45). However, this difference is most likely due to the limited number of trials evaluated in this review compared to the large sample of implants assessed by Chrcanovic et al.

On the other hand, the data analysis of implant survival in this review between the different studies would support the findings of Chrcanovic et al. that survival rates tended to be higher in the delayed implant group compared to the immediate implant group. The survival of implants in the meta-analysis tended to vary between 90 and 95% for immediately placed implants as compared to 97–100% for delayed implant placements, which is similar to the data analysis of this review. Caution is required when interpreting the findings of Chrcanovic et al., as excess inter-study heterogeneity was discovered (*p* = 0.02). Due to a lower quality of evidence being included in the meta-analysis of the 2015 publication, the overall outcome estimate for implant survival may be incorrect [12].

The authors of the 2015 publication also included some non-comparative studies, thus increasing the risk of bias. In addition, the meta-analysis from Chrcanovic et al. was statistically insignificant for the difference in implant survival when implants were placed in the maxilla compared to the mandible, which suggests that the initial analysis was potentially subject to bias and not a reliable assessment (maxillary region; risk ratio 1.61; 95% CI 0.97, 2.66, *p* = 0.07; and mandibular region; risk ratio 2.15; 95% CI 0.62, 7.47, *p* = 0.23). However, the 2015 authors did a further meta-analysis on the overall impact of delayed versus immediate implant placement based on randomised and comparative trials, which supported the original meta-analysis (RR 2.27; 95% CI 1.57, 3.29, *p* < 0.01) that there is an increased risk of failure in fresh extraction sites. Should the studies included in this review have supplemented the meta-analysis of Chrcanovic et al. [12], it is unlikely that the risk ratio effect size would have altered significantly given the low number of studies and small implant sample size of the additional studies observed in this review.

This review was unable to gain any clear data regarding any differences in implant survival between implants placed in the maxilla and the mandible, which has previously been discussed in various trials and evaluated by Chrcanovic et al. (2015). In this review, one study (Bömicke et al., 2017) described implants placed in only the mandibular region, where the survival ranged between 94.7 and 100%, whereas two other studies focusing on implants in solely the maxilla showed the survival rates to be 90.9 and 100% (Esposito et al., 2015; S. Raes et al., 2018). Therefore, the evidence in this review suggests that implant failure may be higher among implants placed in the maxilla, as the lower survival rate was at 90.9% in comparison to 94.7% for the mandible. In the meta-analysis of Chrcanovic et al. (2015), the sensitivity analyses for maxillary- and mandibular-placed implants were statistically insignificant in terms of implant failure. However, the analysis for studies of implants placed in the maxilla trended towards a significant difference (*p* = 0.07). This supports the theory that there is a higher survival rate for implants placed in the mandible, as identified in this review. The higher rate of failure of implants placed in the maxilla is perhaps due to the lower density of bone, which limits primary stability [36]. In the realm of dental implantology, following extraction, an implant may undergo osseointegration and subsequently manifest a periodontal pocket. Over time, this can lead to predictable complications, albeit without necessarily resulting in implant failure. The placement of post-extraction implants at the molar and anterior sectors is contingent upon the immediate application of a provisional crown or the construction of a Single Screw Abutment (SSA). When juxtaposed with post-extraction implants that are merely fitted with a healing screw, especially in instances where an unprotected bone graft has been administered, the latter approach appears to present potential vulnerabilities. It is imperative to delve deeper into these observations and their potential implications for a comprehensive understanding.

The impact of immediate and delayed implant placement upon crestal bone loss was also measured in this review among three studies [25,29,31], with losses of 1.5 mm in the first year following implant placement being considered within normal limits [37] (Kim et al., 2015). The studies included in this review found that crestal bone losses were unfavourable for the immediate implant placement group as compared to the delayed placement groups; given that the losses were significantly different but the extent of loss at 12 months tended not to exceed the accepted 1.5 mm threshold, the findings were not considered clinically meaningful. However, the previous literature contradicts the findings of this review with some authors revealing crestal bone loss among both immediate and delayed implant placements more than 1.5 mm at or prior to 12 months post-surgery [38,39,40]. Therefore, there is uncertainty about whether immediate or delayed implant placement causes any significant difference in crestal bone losses with time. Finally, this review revealed that there were no significant differences in implant stability, as measured using the ISQ, between the immediate and delayed implant groups. This has been supported by the previous literature, which again validates the long-term results and successes of the advances in implantology that have occurred over the past few decades [39]. Additionally, the level of heterogeneity across the included studies was low (I^2^ = 0%) and thus the *p*-value indicated that inter-study heterogeneity was not statistically significant (*p* = 0.60).

In conclusion, the meta-analysis, along with the low heterogeneity between studies, indicates a high level of confidence and certainty in the reported outcome effects, with minimal biases detected in the included studies.

## 5. Conclusions

- Current evidence suggests that there is no statistically significant difference between immediate and delayed implant placement in terms of implant survival or other complications. However, delayed placement may offer a slight survival benefit in selected cases, such as patients with other existing dental and medical comorbidities
- More studies of high-quality randomized trials are needed to measure the impact of immediate versus delayed implant placement upon implant survival. Additionally, research should assess other patient-valued outcomes, including aesthetics, function, and psychological wellbeing.
- Additionally, a body of qualitative evidence is needed to explore the experiences and views of patients who have received dental implants, to identify the factors perceived to influence implant longevity and to recognise the different reasons influencing hygiene compliance. This research could help inform ongoing implant health campaigns and hygiene practices, aiming to improve oral health and potentially reduce the risk of implant failures in patients who have received immediate implant placement.
- Furthermore, a literature review is needed to assess the comparative impact of immediate versus delayed implant placement on the survival of implants within full arch prostheses. Previous research, including this review, has primarily focused on evaluating these interventions for single implants.

## Figures and Tables

**Table 1:** Derivation of review question using PICO framework.

PICO	
Population	Adults aged ≥18 years
Intervention	Immediate dental implant placement in fresh sockets at the time of extraction of the tooth/teeth
Comparator	Delayed dental implant placement in healed sites at least two months after extraction
Outcomes	Survival rate of the implant, in terms of the implant still being present in the mouth at the time of examination

**Table 2:** Summary of search strategy.

PICO	Population	Intervention	Comparator	Outcomes
Search Terms (truncation)	1-Adult 2-Dental implant	3-Immediate placement	4-Delayed placement	5-Survival 6-Failure

**Table 3:** Inclusion and exclusion criteria.

Study Characteristics/PICO	Inclusion Criteria	Exclusion Criteria
Research Design	Randomised controlled trials and non-randomised comparative observational studies	Case-control studies Case studies and series editorials
Publication Date	2014–2022	Before 2014
Language	English	Other languages
Peer-review	Yes	No
Population	Adults aged ≥18 years	Children aged less than 18 years
Intervention	Immediate placement of implants defined as implant placement at the same time as dental extraction	N/A
Comparator	Delayed placement of implants defined as implant placement following soft-tissue and bone healing of dental extraction sockets	N/A
Outcomes	Implant survival and other outcomes, such as complications and bone loss.	Outcomes of little relevance to the review question.

**Table 4:** Summary of evidence characteristics.

Study	Design	Setting	Participants and Type of Rehabilitation	Participants Age	Follow-Up Period	Gap between Extraction and Placement (Delayed Group)	Implant Survival Rate (Immediate vs. Delayed)
Bömicke et al. (2017) [24]	Randomised controlled trial	Germany	Adults requiring implants in posterior mandible (n = 38) one-piece implants OPI (19 participants, OPI group) or two-piece implants TPI (19 participants, TPI group)	21–76 years	3 years	Not reported	94.7% vs. 100%
Cucchi et al. (2017) [25]	Randomised controlled trial	Italy	Adults needing single extraction and implants in posterior maxillary and mandibular regions (n = 92) tapered double-lead threads single implants	20–79 years	1–3 years	3 months	95.9% vs. 100%
Esposito et al. (2015) [26]	Randomised controlled trial	Italy	Adults requiring implants in maxillary second premolar to second premolar region following single-tooth extraction (n = 106) single implant at least 7 mm long with a 4 mm diameter	28–72 years	1 year	4 months	96.3% vs. 100%
Hakobyan et al. (2020) [27]	Non-randomised comparative study	Armenia	Adults with implants in various upper and lower and anterior and posterior sites (n = 52) single implants	26–43 years	5 years	3–5 months	97.8% vs. 98.1%
Han et al. (2016) [28]	Prospective cohort study	South Korea	Patients with implants mostly in the posterior region (85.5%) and predominantly in the mandible (82.3%)(n = 39, Tapered implants featuring a nanostructured calcium-incorporated surface were placed and loaded immediately. The prosthetic restorations comprised single crowns, fixed partial dentures and fixed full arches	18–75 years	1 year	At least 4 months	100% vs. 96.9%
Malchiodi et al. (2016) [29]	Randomised controlled trial	Italy	Adults with implants in maxillary and mandibular premolar and molar sites (n = 40)	35–75 years	1 year	12 weeks	100% vs. 100%
S. Raes et al. (2018) [9]	Prospective cohort study	Belgium	Patients with implants in anterior maxillary region (n = 39), single implants	22–68 years	8–10 years	3 months	93.8% vs. 100%
Slagter et al. (2016) [30]	Randomised controlled trial	Netherlands	Patients with implants in the aesthetic zone (n = 40), single implants	18–72 years	1 year	3 months	100% vs. 100%
Tonetti et al. (2017) [31]	Randomised controlled trial	Italy	Adults with single-tooth extraction and implants in anterior and premolar regions (n = 124), single implants	50–55 years (mean group ages)	1 year	12 weeks	98.3% vs. 100%
Woods et al. (2019) [32]	Retrospective cohort study	Australia	Adults with implants in mandibular and maxillary regions placed due to heck and neck surgery (n = 20) single implants	18–91 years	2–140 months	Not reported	97.4% vs. 90.5%

**Table 5:** Summary of implant survival studies.

Study	Patient Selection	Definition for Implant Survival	Periodontal Probing Pocket Depths (Mean)	Marginal Bone Loss (Mean)	Crestal Bone Loss (Mean)	ISQ	Other Complications	Observations
Bömicke et al. (2017) [24]	Patient selected from hospital department needing a single-tooth implant in the posterior mandible, non-smokers, with minimum 6 mm bone width and 12 mm bone height. Patients randomly assigned immediate or delayed treatment.	Implants which were not mobile or requiring removal.	At 3 years: - Immediate: 2.75 mm - Delayed: 2.98 mm	At 3 years: - Immediate: 1.34 mm - Delayed: 0.67 mm	N/A	N/A	Prosthesis failure was 15.8% in immediate group and 31.3% in the delayed group.	All patients received oral antibiotics 1 h pre-op and 7 days post-op.
Cucchi et al. (2017) [25]	Over two years, patients selected required tooth extraction, had sufficient bone to accommodate a 3.7 × 10 mm implant without grafting and had natural dentition occluding on opposing jaw. Patients required to understand treatment and commit to follow-up.	Implants functional and under load at the 1- or 3-year mark.	At baseline: - Immediate: 3.2 ± 1.3 mm - Delayed: 2.9 ± 1.4 mm No variations seen at follow-up	N/A	At placement: - Immediate: 0.8 ± 0.4 mm - Delayed: 1.2 ± 0.6 mm At follow-up: - Immediate: 1.2 ± 0.6 mm - Delayed: 0.9 + 0.4 mm	At placement: - Immediate: 63.9 ± 12.6 - Delayed: 72.8 ± 9.7	No prosthetic complications.	Both implants that failed were 4.8 mm × 10 mm and placed immediately in maxillary molar regions.
Esposito et al. (2015) [26]	Patients require at least one implant in maxillary region between second premolars and had enough bone to accept 4 × 7 mm implant. Smokers were included.	Implants still present and not showing any mobility or fracture.	N/A	At 1-year follow-up: - Immediate: 0.23 mm - Delayed: 0.29 mm	N/A	N/A	Eight minor complications observed in immediate placement group and one observed in delayed group.	Both immediate and delayed groups had the same number of smokers. Two implant failures occurred in immediate group; in both cases, no grafting was carried out.
Hakobyan et al. (2020) [27]	Patients chosen between 2016 and 2020 and aged between 26 and 43 years who required a dental implant.	No specific details given.	N/A	N/A	After 24 months: - Immediate: 1.06 ± 0.25 mm - Delayed: 1.02 ± 0.29 mm	At placement: - Immediate: 65.2 - Delayed: 68.3	No other complications reported.	Ankylos dental implants were placed in all patients, either 3.75 or 4.25 mm between 10 and 13 mm length
Han et al. (2016) [28]	Healthy patients were selected between 2012 and 2014 who require dental implant(s). Heavy smokers of over 10 cigarettes per day excluded from study.	Implants not lost.	N/A	N/A	N/A	At 1 year: - Immediate: 80.1 - Delayed: 80.5	No other complications reported.	One implant failed, which was in a delayed site in the posterior maxilla. No further details given.
Malchiodi et al. (2016) [29]	Patients selected between 2012 and 2014 who require dental implant(s), with at least 9 mm bone height in the maxilla and 11 mm in the mandible. Patients randomly grouped; 20 implants per group.	Implants which were still present and did not show any mobility, peri-implant bone resorption or infection and no pain.	N/A	N/A	After 12 months: - Immediate: 0.68 ± 0.43 mm - Delayed: 0.40 ± 0.26 mm	At placement: - Immediate: 61.90 ± 9.99 - Delayed: 66.00 ±8.25	Insertion torque higher for delayed group (46.0 nm) compared to immediate group (52.0 nm).	All patients given pre-op chlorhexidine rinse and received antibiotics 1 h pre-op and 6 h post-op.
S. Raes et al. (2018) [9]	Non-smoking patients, with good oral hygiene were referred for implants in anterior maxilla. Patients with diabetes mellitus excluded.	Implants present at follow-up.	At 8 years: - Immediate: 2.7 ± 0.5 mm - Delayed: 3.4 ± 1.7 mm	At 1 year: - Immediate: 1.01 ± 1.73 mm - Delayed: 0.42 ± 1.23 mmAt 8 years: - Immediate: 0.98 ± 1.71 mm - Delayed: 0.49 ± 1.89 mm	N/A	N/A	38% of all patients experienced one or more complications. Abutment screw loosening occurred in 8% of all patients. No significant differences between groups.	As the follow-up period was up to 8 years, there was a higher attrition rate than other studies.
Slagter et al. (2016) [30]	Patients selected between January 2010 and January 2020 who require one implant. Patients excluded if they were smokers, had insufficient bone, periodontal disease or significant vertical bone detection present.	Implants which are functional 1 year after definitive crown placement.	At 12 months: - Immediate (mm): Mesial—3.3 ± 0.7 Distal—3.5 ± 0.8 Buccal—3.2 ± 0.8 Palatal—2.7 ± 0.6 - Delayed (mm)Mesial—3.6 + 0.8 Distal—3.8 + 0.7 Buccal—3.3 + 0.7 Palatal—3.1 + 0.5	At 12 months: - Immediate: 0.56 ± 0.39 mm (mesial), 0.74 ± 0.51 mm (distal) - Delayed: 0.51 ± 0.43 mm (mesial),0.54 ± 0.45 mm (distal)	N/A	N/A	Aesthetic outcome for immediate implants more favourable than delayed implants. PES score for immediate: 15.8 ± 2.1, compared to delayed: 15.3 ± 2.0	All patients given prophylactic antibiotics to take for 7 days prior to surgery.
Tonetti et al. (2017) [31]	Patents required dental implant treatment, were non-smokers or smoked less than 20 cigarettes per day, had no relevant medical conditions and had good periodontal status. Patients offered to be part of study on a sequential basis.	Implants present at follow-up.	At 12 months: - Immediate: 4.1 ± 1.2 mm - Delayed: 3.3 ± 1.1 mm	N/A	Immediate implants showed greater residual bone loss compared to delayed implants, by a mean difference of 0.8 mm.	N/A	Wound failure was 5 times more likely in immediate implant placements than delayed.	Patients given pre-op antibiotics before surgery and diclofenac.
Woods et al. (2019) [32]	Patients included those with cancers of the head and neck, thus having implants as part of rehabilitation treatment. Patients chosen were motivated and had good oral care.	Implants that did not have to be removed due to failure, mobility, or infection.	N/A	N/A	N/A	N/A	Survival rate was lowest amongst patients in the PORT group (post-operative radiotherapy patients)	20% of Straumann SLA Active implants failed. Neoss implants had a 6.1% failure rate.

**Table 6:** Summary of quality judgements.

ROBINS-I							
Non-Randomised Studies	Confounding	Subject Selection	Classification of Interventions	Protocol Deviations	Missing Data	Outcome Measurement	Reporting
Hakobyan et al. (2020) [27]	Unclear	Unclear	Low	Low	Low	Low	Low
Han et al. (2016) [28]	Moderate	Low	Low	Low	Low	Low	Low
S. Raes et al. (2018) [9]	Moderate	Low	Low	Low	Moderate	Low	Low
Woods et al. (2019) [32]	Moderate	Low	Low	Low	Low	Low	Low
ROB 2.0							
Randomised Trials	Randomisation	Protocol Deviations	Missing Data	Outcome Measurement	Reporting	-	-
Bömicke et al. (2017) [24]	Unclear	Low	Low	Low	Low	-	-
Cucchi et al. (2017) [25]	Low	Low	Low	Low	Low	-	-
Esposito et al. (2015) [26]	Unclear	Low	Low	Low	Low	-	-
Malchiodi et al. (2016) [29]	Some concern	Low	Low	Low	Low	-	-
Slagter et al. (2016) [30]	Unclear	Low	Low	Low	Low	-	-
Tonetti et al. (2017) [31]	Low	Low	Low	Low	Low	-	-
